# Clinical aspects of a nationwide epidemic of severe haemolytic uremic syndrome (HUS) in children

**DOI:** 10.1186/1757-7241-19-44

**Published:** 2011-07-28

**Authors:** Lars Krogvold, Thore Henrichsen, Anna Bjerre, Damien Brackman, Henrik Dollner, Helga Gudmundsdottir, Gaute Syversen, Pål Aksel Næss, Hans Jacob Bangstad

**Affiliations:** 1 Department of Paediatrics, Oslo University hospital, Ulleval, Kirkeveien 166, 0407 Oslo, Norway; 2 Department of Paediatrics, Section for Specialised Medicine, Oslo University Hospital, Rikshospitalet, Norway; 3 Department of Paediatrics, Haukeland Hospital, Bergen, Norway; 4 Children's Department, St. Olavs University Hospital of Trondheim, Institute of Laboratory Medicine, Children's and Women's Health, Norwegian University of Science and Technology, Trondheim, Norway; 5 Nephrological Department, Oslo University Hospital, Ulleval, Norway; 6 Department of Microbiology, Oslo University Hospital, Ulleval, Norway; 7 Department of Paediatric Surgery, Oslo University Hospital, Ulleval, Norway

## Abstract

**Background:**

Report a nationwide epidemic of Shiga toxin-producing E. coli (STEC) O103:H25 causing hemolytic uremic syndrome (D+HUS) in children.

**Methods:**

Description of clinical presentation, complications and outcome in a nationwide outbreak.

**Results:**

Ten children (median age 4.3 years) developed HUS during the outbreak. One of these was presumed to be a part of the outbreak without microbiological proof. Eight of the patients were oligoanuric and in need of dialysis. Median need for dialysis was 15 days; one girl did not regain renal function and received a kidney transplant. Four patients had seizures and/or reduced consciousness. Cerebral oedema and herniation caused the death of a 4-year-old boy. Two patients developed necrosis of colon with perforation and one of them developed non-autoimmune diabetes.

**Conclusion:**

This outbreak of STEC was characterized by a high incidence of HUS among the infected children, and many developed severe renal disease and extrarenal complications. A likely explanation is that the O103:H25 (*eae *and *stx_2_*-positive) strain was highly pathogen, and we suggest that this serotype should be looked for in patients with HUS caused by STEC, especially in severe forms or outbreaks.

## Background

Haemolytic uremic syndrome (HUS) is a severe, acute and dramatic disease affecting previously healthy children. HUS is defined as a triad of acute kidney injury, microangiopatic haemolytic anaemia and thrombocytopenia in patients with no other explanation for coagulopathy [1] e.g. thrombotic thrombocytopenic purpura. More than 90% of the cases are due to Shiga toxin-producing *E. coli *(STEC) infections; termed typical HUS or diarrhoea associated HUS (D+HUS). Many different serotypes can cause HUS, the most prevalent in Europe and USA being O157:H7 [2,3]. A broad spectrum of extrarenal complications may occur in HUS, the most common are gastrointestinal and cerebral. Extrarenal involvement at an early stage is associated with increased morbidity and mortality. Although several epidemics, caused by O157 [4] and other serotypes [5] have been reported, the majority of HUS cases appear sporadic or in small clusters [1].

In 2006 a nationwide outbreak of STEC-infections took place in Norway. Totally 17 cases (16 children and one adult) were identified during the outbreak, all caused by a rare variant (O103:H3, *eae *and *stx_2_*-positive). Some microbiological, serological and epidemiological aspects of the outbreak have previously been reported [6,7]. In this article we will focus on those children that presented with typical HUS since the clinical course was characterized by an aggressive disease with significant extrarenal complications.

## Methods

Within a short period of time from 30^th ^of January to 13^th ^of March 2006 a nationwide outbreak of STEC -infections occurred. As soon as the epidemic pattern was confirmed, health personnel were informed by the National health authorities, and instructed to collect faecal samples on all suspected cases with possible HUS-related *E. coli *infection (diarrhoea and fever). At the same time microbiological laboratories were instructed to investigate specifically for serotype O103.

Clinical data of the children were retrieved from medical notes and charts. All five university-hospitals in Norway treating children with HUS were contacted, and a review of all admissions were done at each department to ensure all cases being included

## Results

Sixteen children were infected by *E. coli *O103:H25 during the outbreak. Ten of these children (six girls and four boys), with a median age of 4.3 years (range 1.8-8.5), were admitted to hospital with the clinical picture of typical D+ HUS and constitute our study population. The children were living widely spread, and were admitted to the department of paediatrics at four different University Hospitals with a median time of symptoms of 5 days (range 2-10).

### Presentation on admission

Eight of the ten children were severely affected with bloody diarrhoea when admitted to hospital. Eight patients were oligoanuric with urine output less than 0.5 mL/kg/h. Elevated serum creatinine, leukocytosis, thrombocytopenia, elevated lactate dehydrogenase (LD) and hyponatremia (nine out of ten) were common findings (Table 1). The haemoglobin values on admission varied, but all patients developed marked haemolytic anaemia during their first week in hospital and received blood transfusion, the indication being respiratory compromise or severe anaemia (Table 1).

**Table 1:** Laboratory values for ten patients with HUS caused by *E. coli *O103:H25

Patient	Haemoglobin		Creatinine		Lactate dehydrogenase		Thrombocytes		Leucocytes	Sodium
**No**	**(g/dL)**		**(μmol/L)**		**(U/L)**		**(× 10^9^/L)**		**(× 10^9^/L)**	**(mmol/L)**

	**Admission**	**Min.**	**Admission**	**Max.**	**Admission**	**Max.**	**Admission**	**Min.**	**Admission**	**Admission**

1	7.6	5.7	461	492	2888	2888	43	29	9.1	132
2	9.3	7.7.	82	162	2241	3575	17	16	7.1	137
3	11.5	7.5	196	407	3888	3888	84	62	33.8	128
4	11.2	6.5	231	358	3827	4287	41	41	16.5	132
5	9.5	7.6	627	673	3803	3803	61	23	33.7	133
6	9.1	7.1	421	658	4036	4036	60	60	21.0	127
7	12.2	5.5	143	422	764	3212	134	67	30.0	132
8	16.9	8.8	107	276	2641	3431	61	35	41.3	121
9	12.5	7.6	153	405	2419	2928	110	66	35.3	127
10	6.1	5.4	84	84	2566	2566	109	107	25.4	125
Median	10.4	7.3	175	406	2764	3503	61	51	27.7	130

HUS: Haemolytic uremic syndrome

Min: Minimal level

Max: Maximal level.

### Renal complications and outcome

Eight patients required dialysis (Table 2). Haemodialysis was chosen in four children, in three cases based on the severity of abdominal pain and activity of enterocolitis and in one case due to recent abdominal surgery. Peritoneal dialysis was chosen in four children with less severe abdominal symptoms on admission. However, in two of these patients intestinal perforation occurred and peritoneal dialysis were therefore switched to haemodialysis. The median time on dialysis was 15 days (3 days - > 1 year), or 14 days (3-34 days) excluded the patient who developed ESRF and later had a kidney transplant. On follow up one year after diagnosis, four patients had regained normal renal function and normal blood pressure, and four patients had low-grade proteinuria and/or microscopic haematuria, but no hypertension, defined as blood-pressure above the 95^th ^percentile for age, sex and height. Patient number 9 developed end stage renal failure and was on dialysis until she received a living related kidney transplant 12 months after her first symptoms.

**Table 2:** Mode of dialysis, acute symptoms and complications in ten patients with D+HUS caused by *E. coli *O103:H25

Patient	Dialysis		Neurological		Gastrointestinal	Others
				
No	Mode		Symptoms	MRI/CT		
		
	Duration (Days)					
1	HD	7	-	-	-	-

2	-	-	-	-	-	-

3	PD	15	Reduced consciousness	MRI: Infarction basal ganglia	-	-

4	PD/HD*	15	-	-	Perforation of colon	-

5	HD	13	Reduced consciousness	CT normal	-	-

6	PD	34	Generalised seizures before admission	MRI normal (8 months after)	-	-

7	HD	14	-	-	Laparoscopy: suspect appendicitis	Insulin for 5 days

8	HD	3	Death due to fatal cerebral oedema	CT/MRI: generalised oedema, infarction of basal ganglia	-	-

9	PD/HD*	∞	Reduced consciousness and seizures	CT/MRI normal	Colon necrosis	Diabetes mellitus

10	-	-	-	-	-	-

*Switched to HD because of intestinal perforation

HUS: Haemolytic uremic syndrome

MRI: magnetic resonance imaging

CT: Computer tomography

HD: haemodialysis

PD: Peritoneal dialysis.

### Extrarenal complications

Five of the children had signs of CNS involvement (Table 2). One boy died three days after admission of cerebral herniation. Cerebral magnetic resonance imaging (MRI) showed generalised oedema and bilateral infarcts in the basal gangliae. Four patients presented cerebral seizures and/or reduced consciousness (Table 2). One of these had a unilateral infarction in the area of putamen on cerebral MRI. He recovered and neurological examination on discharge was completely normal. The other patients with cerebral symptoms had normal MRI-findings.

Appendectomy was performed in one girl in a local hospital before the HUS diagnosis was established. The removed appendix was not inflamed. Two patients developed necrosis of colon with perforation and underwent laparotomy on hospital day 6 (left hemicolectomy) and day 24 (subtotal colectomy), respectively. One patient developed permanent insulin dependent diabetes mellitus with negative anti-GAD antibodies, and another had transient hyperglycaemia with the need of insulin-infusion for five days.

### Antibiotics

Seven patients were treated with antibiotics. In patient 6 antibiotics was started at the local hospital because of suspected sepsis 6 hours prior to the diagnosis of HUS, although presenting with bloody stools, anuria and thrombocytopenia (Table 1). The remaining six children who received systemic antibiotics, all started treatment at least three days after the diagnosis of HUS was established. In three children (patients 3, 4 and 9) antibiotics were administered in the peritoneal dialysis fluid on the assumption of peritonitis. Bacterial cultures were later proven negative. Five patients were given antibiotics intravenously, for suspected sepsis (patient 2, 8 and 9), perforation of colon (patient 4 and 9) or catheter related infection (patient 5), respectively.

### Microbiology

In eight of ten patients who developed HUS, specific IgG antibodies against O103 were detected. In four of the patients, O103:H25 was found in faecal samples. The bacteria were also found in faecal samples from six children who did not develop HUS. In one boy (patient 1) faecal samples could not be collected, and IgG antibodies against O103 were not detected. We have included this patient in the report based on the fact that he had eaten the specific smoked sausage and was the first reported case in the outbreak [6].

## Discussion

We present a nationwide outbreak of STEC causing severe HUS in a high percentage of the affected children. The clinical course was characterized by an aggressive disease with significant extrarenal complications. In Norway there are five University Hospitals with paediatric departments, all in close contact with the local paediatric departments. Due to the alert of the outbreak, the University Hospitals were contacted to treat all cases of HUS. The affected children were admitted to four of these departments, the 5^th ^department confirming that no patient with HUS was admitted during the outbreak. Therefore we conclude our material includes all the affected children.

Several clinical and biochemical features at onset of HUS have been proposed to be related to poor prognosis [8]. Among the most often proposed factors are leukocytosis and anuria [9-11]. A case-control study from 2006 dealt with 17 deaths among patients with HUS and concluded that those presenting with oligoanuria, dehydration, WBC > 20 × 10^9^/L and haematocrit > 23% are at substantial risk of fatal HUS [12]. Most of the patients in our material (seven of 10) had white blood count above 20 × 10^9^/L on admission; the highest level (41.3 × 10^9^/L) was registered in the boy who died. He also had the highest haemoglobin-level and thereby haematocrit, corresponding well with the risk-factors pointed out by Oakes et al. [12]. Eight of ten patients were oligoanuric on admission.

Seven of the children needed transient dialysis, with a median duration of 15 days. One patient developed end stage renal failure and received a living related kidney transplant one year later. According to the literature, around half of children with HUS will need dialysis, with a median duration of 5 to 7 days [13]. This epidemic shows a higher proportion of patients developing a very severe disease with extrarenal complications.

CNS involvement is common and is reported in 20-50% of HUS cases [14,15] and was present in five patients in our material. Common signs of CNS involvement in HUS are seizures, reduced level of consciousness, hemiparesis, visual disturbances and brain stem symptoms. Basal ganglia involvement is a typical MRI-finding in HUS-patients with neurological complications [15], and was present in two of our patients (Table 2).

The reported incidence of colon necrosis and perforation in case studies varies from 1-8% [16-19]. A review by Siegler in 1994 reported a total incidence of colon necrosis/perforation at 2% [14]. Two of the patients in the present study developed necrosis of colon (20%). Patient 9 underwent subtotal colectomy 27 days after onset of symptoms. In a paper reviewing the occurrence of colonic necrosis in patients with HUS, a mean of 11 days after onset of symptoms was reported [18]. Both our patients were on peritoneal dialysis when the necrosis occurred. To our knowledge peritoneal dialysis being a risk factor for the development of colonic necrosis in patients with HUS has not been reported. However, peritoneal dialysis may mask abdominal symptoms leading to delay in diagnosis and surgical treatment.

Diabetes mellitus is a rare complication of HUS and mainly occurs in severe cases [19]. A systematic review of 21 studies concluded a pooled incidence of 3.2% [20]. Autopsy studies have shown thrombosis of the vessels supplying the islets of Langerhans with preservation of the exocrine pancreas [21]. One girl (patient 9) developed permanent insulin-dependent diabetes mellitus. There was no evidence of autoimmune diabetes as all diabetes related autoantibodies were negative. She was seriously ill on admission and developed necrosis of colon and end stage renal failure, and finally received a kidney transplant. This corresponds to a previous review, stating that children with HUS who develop diabetes mellitus, were more likely to have severe disease with increased mortality risk [20]. Among survivors, 38% were left with permanent diabetes requiring insulin [20]. Even though this patient also needed a kidney transplant, simultaneous pancreas and kidney transplantation was not an option, due to our policy to use living related donors which favourably influence outcome.

All children received blood transfusions. The mean haemoglobin-value at transfusion was 6.9 g/dL. Erythrocyte transfusions in HUS should be avoided if possible, and some suggest it is indicated only when haemoglobin is below 6.0 g/dL [22]. Nevertheless, the usual indications for erythrocyte transfusions apply, i.e. respiratory compromise and cerebral involvement, and 70-80% of patients with HUS will require transfusions [1,23].

Antibiotics is contraindicated in the treatment of possible STEC infections, due to increased toxin-release from bacterial lysis [24] or increased production of toxin due to induction of bacteriophages on which *stx*-genes are located [25]. In our material, six children received intravenous antibiotics. However, the treatment was initiated after the diagnosis of HUS was established in five, and none of the patients had antibiotics started as treatment of HUS, but on the suspicion of secondary bacterial infections.

To our knowledge this is the first outbreak of HUS caused by *E. coli *O103. The microbiological, serological and epidemiological aspects of the outbreak have previously been published [6,7]. We found positive faecal samples for O103:H25 in ten children during the outbreak, and four of these developed HUS. This high incidence of HUS among the infected patients contrasts previous reports on *E. coli *O157:H7 outbreaks, in which 11%-14% developed HUS [26,27]. During the present outbreak, the attention-level in the population was kept high due to huge interest of the epidemic in the media. National health authorities instructed parents to see a physician if their child had any symptoms of diarrhoea or vomiting. Physicians were informed by the Norwegian Institute of Public Health to collect faecal samples from children with diarrhoea and the number of faecal samples analyzed by the microbiological laboratories increased. On that background it is unlikely that the number of children infected by this specific O103-strain was substantially higher than those diagnosed. The specific diagnosis of O103 was confirmed either through faecal sampling or serology in nine out of ten patients. This corresponds to Lynn et al. who found that 84% of the cases of HUS in UK and Ireland in 1997-2001 were similarly confirmed [2]. In the present report positive faecal samples were found in only four of the patients with HUS. The explanation to this might partially be due to difficulties collecting adequate samples; several of the children did not pass stool for several days after admission to hospital. In a prospective surveillance of Canadian children with HUS from 2000 to 2002, stool cultures showed evidence of bacterial pathogens in 67% of the patients, but only two non-O157-strains were found [28].

## Conclusion

This outbreak of colitis caused by STEC serotype O103:H25 (*eae *and *stx_2_*-positive) was characterized by a very high incidence of HUS, and the majority of the affected children experienced severe renal disease and significant extrarenal complications. Although genetic variability theoretically could explain Norwegian children being more prone to severe disease, we suggest that STEC serotype O103:H25 (*eae *and *stx_2_*-positive) may be highly pathogenic and should be investigated for in future HUS outbreaks.

## List of abbreviations

HUS: haemolytic uremic syndrome; D+: diarrhoea associated; STEC: shiga-toxin-producing E. Coli; EHEC: enterohaemorrhagic E. Coli; LD: lactate dehydrogenase; MRI: magnetic resonance imaging; stx: shigatoxin; WBC: white blood cells.

## Competing interests

The authors declare that they have no competing interests.

## Authors' contributions

All authors have read and approved the final manuscript. LK contributed during all steps in designing and producing this report, coordinated the author team, been involved in the treatment in most of the patients, and controlled data sampling and -analysis. TH was deeply involved in the medical treatment of 50% of the patient included in this report, contributed essentially to the writing of the manuscript, and reviewed current literature. AB, DB, HD took medical care of 50% of the patients, participated in designing the report, provided data, contributed to analyzing the data and reviewed critically the manuscript in all stages of the process. HG performed dialysis in patients with HUS, reviewed up to date literature in the field of treatment of HUS, reviewed data and read all versions of the manuscript and gave comments on all sections of the manuscript, especially to the discussion. GS contributed with knowledge and competence in detecting and analysing microbiological data controlled all microbiological data and outlined the section "methods" and contributed to the description of the microbiological results and the discussion. PAN was involved in the surgical treatment of many of the patients included, contributed essentially to the writing of the manuscript. HJB is the supervisor of the first author, and contributed in all parts in the process of making this article.
